# Nutrition environments in early childhood education: do they align with best practice?

**DOI:** 10.1017/S136898002400096X

**Published:** 2024-04-29

**Authors:** Anna Aristova, Alison C Spence, Christopher Irwin, Audrey Elford, Laura Graham, Penelope Love

**Affiliations:** 1 School of Exercise and Nutrition Sciences, Deakin University, Waurn Ponds, VIC 3216, Australia; 2 Institute for Physical Activity and Nutrition (IPAN), School of Exercise and Nutrition Sciences, Deakin University, Geelong, VIC, Australia; 3 School of Health Sciences and Social Work (SHS), Nutrition and Dietetics, Griffith University, Southport, QLD, Australia

**Keywords:** Nutrition policy, Policy assessments, Early childhood education, Nutrition environments, Long day care, Early childhood nutrition

## Abstract

**Objective:**

To assess the comprehensiveness (scope of nutrition guidance) and strength (clarity of written language) of centre-based nutrition policies (CBNP) within early childhood education (ECE) centres. To also consider the applicability of an existing CBNP assessment tool and policy alignment with best practice food provision and feeding practices.

**Design:**

Cross-sectional online study to assess written ECE CBNP using the Wellness Child Care Assessment Tool.

**Setting:**

Licenced ECE centres in the state of Victoria, Australia.

**Participants:**

ECE centres (operating at least 8 h per d, 48 weeks per annum), stratified by location (rural and metropolitan), centre management type (profit and not-for-profit) and socio-economic area (low, middle, high).

**Results:**

Included individual CBNP (*n* 118), predominantly from metropolitan centres (56 %) and low-medium socio-economic areas (78 %). Policies had low overall Wellness Child Care Assessment Tool scores, particularly strength scores which were low across all four domains (i.e. nutrition education, nutrition standards, health promotion and communication/evaluation). The nutrition standards domain had the lowest strength score. The communication/evaluation domain had the lowest comprehensiveness score. Content analysis indicated low scores may relate to the Wellness Child Care Assessment Tool applicability for the Australian context due to differences in best practice guidance.

**Conclusion:**

Despite the presence of written nutrition policies in ECE centres, many showed weak language and lacked comprehensiveness and strength. This may relate to poor implementation of best practice food provision or feeding practices. Low scores, however, may partly stem from using an assessment tool that is not country-specific. The redevelopment of country-specific tools to assess ECE CBNP may be warranted.

Early childhood is an important developmental period where lifelong dietary habits are formed^(1)^. Studies indicate that dietary behaviours established during early childhood track into adulthood,^(2)^ with the transition from breastfeeding to autonomous food selection influencing short-term and long-term health outcomes^(3)^. Birth to age 5 years is a crucial life stage where dietary patterns and food preferences emerge and are shaped^(4,5)^. Ensuring a nutrient-rich diet at this stage of rapid growth and development will support postnatal brain development, cognitive development, bone health and oral health^(1,6)^. Furthermore, poor diet quality can negatively impact mental health^(7)^, dental health^(6)^ and energy levels in children^(8)^. Healthy eating behaviours may also reduce the prevalence of obesity in adulthood, given the aetiology of obesity across the lifespan^(9)^. Intervening early can therefore foster early establishment of healthful dietary patterns whilst aiming to reduce the prevalence of adverse health effects from poor nutrition before food preferences become internalised^(1,3)^.

Early childhood education (ECE) is an important setting to explore food provision for children under the age of 5 years, with high ECE uptake across high-income countries^(10,11)^. In Australia, approximately 60 % of children attend ECE in the form of long day care (hereafter referred to as childcare), defined as centre-based care for children up to age 6 years, operating for at least 8 h/d, 48 weeks/year. Australian children spend an average of 31 h/week in childcare (i.e. 3–4 d/week), consuming 40–67 % of their daily dietary intake in these settings^(12)^. Research shows food intakes of children in childcare are inconsistent with dietary recommendations, with low intakes of vegetables, fruit and whole grains and high intakes of saturated fat and sweet snacks^(13,14)^. Therefore, it is apparent that childcare settings can make an important contribution to child nutrition through the quantity and quality of food provided to children.

Nutrition interventions to improve food provision and intake of children within childcare settings have achieved small but significant improvements^(15)^. Interventions targeting the nutrition environment (e.g. using a centre-based nutrition policy (CBNP)) have been found more effective than those focusing on individual behaviour change (e.g. providing information to parents)^(16–18)^. The role of a well-structured and concise policy document is essential to most organisations in any setting as it provides guidance and procedures to ensure smooth day-to-day operations. For childcare settings, such streamlined nutrition guidance is critical to creating a positive nutrition environment. In Australia, childcare centres are required to comply with the National Quality Framework with seven National Quality Standards aimed at improving health outcomes in children^(19)^. Developed by the Australian Children’s Education and Care Quality Authority (ACECQA), which provides national support for Australian childcare centres, these standards provide generic guidelines to assist ECE services in aligning their CBNP to best practice,^(19)^ encouraging nutritious food provision that considers children’s nutritional, development and cultural needs and is adequate in quantity. Under these guidelines, it is a requirement that approved providers not only have policies in place for nutrition, food and beverages, but they also need to ensure implementation of these policies. The national Get Up and Grow guidance document, which is a series of online resources intended to support ECE services with their food provision and feeding practices, is also available to centres to assist them in achieving the National Quality Standards food provision standard; however, interpretation and application of this document vary across Australian states and territories^(20)^. The varied interpretation across jurisdictions creates challenges to operationalise the National Quality Standards and achieve healthy food provision and has resulted in jurisdictions developing their own food provision guidelines and resources^(20)^.

Additional challenges in developing and adopting effective nutrition policies may stem from socio-economic differences and geographical location. This study delves into the well-documented influence of socio-economic disparities on health behaviours, prompting an investigation into how socio-economic status and rurality could influence the quality of nutrition policies^(21)^. It is posited that lower socio-economic status and rural settings might impact policy comprehensiveness and effectiveness due to associated challenges, such as limited health literacy and resources^(21,22)^.

Research to date predominantly reports on the existence or absence of childcare CBNP, with few assessing constructs of comprehensiveness and strength of the policy content to guide best practice food provision and feeding behaviours. The comprehensiveness and strength of policies are an important consideration to ensure the content covers a large scope of nutrition guidance using language that can be understood and applied by the end user/s (i.e. childcare staff). Assessing childcare CBNP can provide crucial insights as to improvements that could further support childcare staff and parents/children who access these services^(23)^. Research involving policy assessment is scarce. Indeed, few studies have been conducted in the USA using the Wellness Child Care Assessment Tool (WellCCAT)^(24)^. One study used the tool to assess policies of New Zealand (NZ) childcare centres^(25)^. Assessment of Australian CBNP strength and comprehensiveness is currently lacking.

This study aimed to assess the comprehensiveness (of content) and strength (of written language) of CBNP in a sample of Australian childcare centres. The applicability of employing WellCCAT for the Australian context was also investigated.

## Methods

### Recruitment

Data collection occurred between July and November 2021. Childcare centres (operating at least 8 h per d, 48 weeks per annum) situated in the state of Victoria (Australia) and listed on the ACECQA website (https://www.acecqa.gov.au/resources/national-registers) as of June 2021 were eligible for inclusion. At the time of study, there were *n* 1802 listed childcare centres across Victoria^(26)^. Childcare centre directors were initially contacted by email and invited to upload written CBNP documents as one component of a survey hosted in Research Electronic Data Capture (REDCap). Due to low participation, likely as a result of coronavirus disease 2019, additional web-based collection of publicly available data was undertaken to access centre characteristic information and CBNP. The websites of Victorian childcare centres that did not participate in the original request were searched by two researchers for publicly available nutrition policies. Policies selected from individual centre websites were nutrition-related policy documents that were of similar format and content to the initial policies supplied by directors. Keywords searched included ‘health policy’, ‘nutrition policy’, ‘food policy’ and ‘wellbeing policy’. Publicly available sample menus or shortened versions of policies were not included as they did not encompass a complete nutrition policy.

### Centre characteristics

Postcodes were used to determine centre location (rural or metropolitan) using the Australian Government Department of Agriculture, Water and the Environment website via the Postcode Classification Search^(27)^. Socio-economic Indexes for Areas (SEIFA) deciles were obtained from the National Quality Standards Childcare data index^(26)^, using the Index of Relative Socio-economic Disadvantage^(28)^. SEIFA deciles were assigned to centres according to their postcode and categorised as low (deciles 1–3) (greatest relative disadvantage), medium (deciles 4–7) or high (deciles 8–10) (least relative disadvantage). Various characteristics of interest were also recorded throughout each policy assessment in an Excel document, such as centre management type (profit or not-for-profit), reference to additional policies (such as a celebrations policy) and reports of flexible or progressive mealtimes.

Summaries and tabulations were used to describe centre characteristics. Prior to all comparative analyses, data were checked for assumptions of normality (QQ plot of model residuals and Shapiro–Wilk test *P* > 0·05) and homogeneity of variance (Levene’s test *P* > 0·05). All data were non-normally distributed; hence non-parametric analyses were employed. Data for comprehensiveness and strength scores were compared according to centre characteristics (i.e. location, management type, SEIFA index). Where these characteristics had more than two categories (i.e. SEIFA index: low *v*. medium *v*. high), differences were explored using the Kruskal–Wallis test, with Dunn’s test post hoc analysis conducted on all significant main effects. Centre characteristics with only two categories (i.e. location: metropolitan *v*. rural; management type: for-profit *v*. not-for-profit) were compared using the Mann–Whitney *U* test. Data for Nutrition standards (NS) strength scores were also found to be zero inflated during pre-analysis data visualisation and assumption checks. As non-parametric tests are not specifically designed to handle zero-inflated data (a substantial portion of the data were zeros – higher than expected under typical distribution), zero-inflated negative binomial regression analyses were used to determine whether scores were influenced by centre characteristics. Where a main effect of the SEIFA index was identified in these analyses, post hoc Tukey tests were conducted for pairwise comparisons. All statistical analyses were performed in R Studio (version 2023·06·1 + 524) using the following packages: ‘ggplot2’, ‘ggpubr’, ‘car’, ‘rstatix’, ‘emmeans’ and ‘pscl’. All data are presented as median and inter-quartile range (IQR) unless otherwise stated. Statistical significance was accepted as *P* < 0·05.

### Policy assessment

Policies were evaluated using the WellCCAT – a validated instrument developed in 2011 for quantitative assessment of nutrition and physical activity statements included in US childcare policy documents^(24,29)^. The comprehensiveness construct of the WellCCAT tool investigates the scope of written nutrition guidance (i.e. the breadth of nutritional content), whilst the strength construct assesses the strength of written language used to describe the nutrition guidance (i.e. clear and specific written language and instructions). As the scope of this study focused on the nutritional aspect of written policies, all questions relating to physical activity were removed. This resulted in a final checklist tool containing forty-six items in total, across four domains: Domain 1, nutrition education (NE: six items); Domain 2, nutrition standards for food and beverage provision (NS: seventeen items); Domain 3, promotion of healthy eating (HP: sixteen items); and Domain 4, communication and evaluation (CE: seven items). The forty-six-item checklist was initially applied independently to three policies by two scorers to check for consistency of interpretation and application prior to evaluating the remaining policies. Uncertainty or disagreement over items between scorers was discussed between both scorers and a moderator to reach a consensus on the interpretation. Each remaining policy was then scored independently by both researchers. To ensure consistency for the remainder of data collection, three online meetings occurred (one after every approximately thirty policy assessments) where each WellCCAT item from each policy was shared to ensure the consistent application was applied. Where scores did not align, items were discussed to reach a consensus score. Scorers and moderators are all nutrition-qualified professionals.

When applied to CBNP, each item within the forty-six-item checklist was allocated a WellCCAT score of 0, 1 or 2 using pre-defined criteria. If a criterion was not addressed in the policy, the item scored a 0; if present but vague/weakly described (e.g. we ‘encourage’), the item scored a 1; and if specifically described using concise and clear wording (e.g. it is ‘required’), the item scored a 2. WellCCAT scores were recorded in a Microsoft Excel document (version 16·49). Results were presented according to how many individual centre policies addressed the item (*n* = number of centre policies) along with the relevant domain and item number. Comprehensiveness and strength scores were calculated for each of the four domains using the method described by Falbe and colleagues^(29)^.

To assess the applicability of the WellCCAT tool for the Australian context, inductive content analysis was conducted whilst scoring the CBNP. Scorers systematically documented their interpretations of recurring issues encountered whilst applying the WellCCAT scoring criteria^(30)^. These reflective notes were thematically analysed to identify patterns regarding the applicability and nuances of the WellCCAT tool within the Australian policy landscape.

## Results

Across the *n* 1802 Victorian Early Childhood Education and Care centres listed on the ACECQA website, only *n* 118 CBNP were available for inclusion in this study, representing a sample of 6·6 % of ACECQA-listed childcare centres in Victoria. This included nineteen policies uploaded by childcare centres and ninety-nine policies that were publicly available from the websites of Victorian childcare centres that did not participate in the original request (*n* 1783). Table 1 outlines the characteristics of the childcare centres included in this study. Most of the childcare centres were situated in metropolitan locations (55·9 %), were ‘not-for-profit’ management type (71·2 %) and were categorised as low or medium SEIFA (73·7 %).

**Table 1:** Characteristics of childcare centres with available nutrition policies (*n* 118)

Characteristics	Number of centres	%
Location		
Metropolitan	66	55·9
Rural	52	44·1
Management type		
For-profit	34	28·8
Not-for-profit	84	71·2
SEIFA index*		
Low	40	33·9
Medium	47	39·8
High	31	26·3
Size of operation†		
Small (≤ 50 children)	32	27·1
Medium (51–150 children)	75	63·6
Large (> 150 children)	11	9·3

* SEIFA: Socio-economic Indexes for Areas, categorised based on postcode using the Index of Relative Socio-economic Disadvantage, where low = deciles 1–3, medium = 4–7, high = deciles 8–10 in accordance with ABS census data (https://www.abs.gov.au/websitedbs/censushome.nsf/home/seifa).

† Maximum number of children per day in the centre.

Table 2 presents nutrition policy WellCCAT comprehensiveness and strength scores for each domain as an overall score and by location, management type and SEIFA index. The overall median comprehensiveness score was 60/100 (IQR = 13; range: 12–72), and the median strength score was 17/100 (IQR = 8; range: 0–31). Across the four domains, the highest median comprehensiveness scores were observed for Domain 1 (nutrition education (NE 83/100; IQR = 16; range: 0–83)) and Domain 3 (health promotion (HP 69/100; IQR = 16; range: 21–94)). Domain 4 CE)) had the lowest median comprehensiveness score (43/100; IQR = 28; range: 0–86) and Domain 2 (NS) had the lowest median strength score (0/100; IQR = 0; range: 0–31)

**Table 2:** Nutrition policy scores for comprehensiveness and strength, by WellCCAT domain, service location, management type and SEIFA index (*n* 118)*,†,‡,§

Domain and scored component			Service location					Management type					SEIFA index						
	Overall score		Metro (*n* 66)		Rural (*n* 52)		*P* value	For-profit (*n* 34)		Not-for-profit (*n* 84)		*P* value	Low (*n* 40)		Medium (*n* 47)		High (*n* 31)		*P* value
	Median	IQR	Median	IQR	Median	IQR		Median	IQR	Median	IQR		Median	IQR	Median	IQR	Median	IQR	
Complete policy																			
Comprehensiveness	60	13	59	17	60	12	0·841	59	12	60	15	0·145	61	13	59	11	60	14	0·219
Strength	17	8	17	10	17	6	0·210	17	8	17	9	0·274	17	10	14	6	18	9	0·062
Domain 1 – Nutrition education (NE)																			
Comprehensiveness	83	16	83	16	83	16	0·786	83	16	83	16	0·709	83	16	83	16	83	16	0·864
Strength	17	33	**17**	**33**	**17**	**17**	**0·021**	17	29	17	33	0·567	17	20	17	17	17	25	0·208
Domain 2 – Nutrition standards (NS)																			
Comprehensiveness	47	18	47	17	44	18	0·972	**41**	**12**	**47**	**14**	**0·007**	47	18	47	18	47	18	0·573
Strength	0	0	0	0	0	0	0·060	0	0	0	0	0·546	**0**	**0**	**0**	**0**	**0**	**0**	**0·049**
Domain 3 – Health promotion (HP)																			
Comprehensiveness	69	16	72	20	69	12	0·439	75	25	69	12	0·468	75	19	69	19	75	12	0·331
Strength	25	12	25	12	25	14	0·574	25	12	25	12	0·840	25	12	25	15	25	12	0·965
Domain 4 – Communication and evaluation (CE)																			
Comprehensiveness	43	28	43	28	43	18	0·462	**43**	**11**	**50**	**28**	**0·042**	43	14	43	28	43	35	0·255
Strength	29	15	29	15	29	15	0·835	14	15	29	29	0·062	**29**	**16**	**14**	**29** ||	**29**	**24**	**0·021**

Data are median (IQR). Data in bold text indicates statistically significant comparisons (*P* < 0·05).

* Overall, comprehensiveness and strength scores were calculated using the *Wellness Child Care Assessment Tool* (WellCCAT)^(29)^.

† Domain refers to the different categories within the WellCCAT analysis (four in total).

‡ *Socio-economic Indexes for Areas* (SEIFA). The Index of Relative Socio-economic Disadvantage was used (low = deciles 1–3, medium = deciles 4–7, high = deciles 8–10). Available at: https://www.abs.gov.au/websitedbs/censushome.nsf/home/seifa.

§ Individual centre SEIFA index obtained from National Quality Standards Data, NQS Data Q4, 2022. Available at: https://www.acecqa.gov.au/nqf/snapsh.

|| Significantly different from low (*P* = 0·049) and high (*P* = 0·034) SEIFA index groups.

A significant difference in NE strength scores was observed based on centre location (W = 2118, *P* = 0·021), with lower scores for rural centres (median = 17/100, IQR = 17; range: 0–33) compared with metropolitan centres (median = 17/100, IQR = 33; range: 0–50). For comparisons based on centre management type, NS comprehensiveness scores were significantly higher (W = 1881, *P* = 0·007) for ‘not-for-profit’ centres (median = 47/100, IQR = 14; range: 12–81) than ‘for-profit’ centres (median = 41/100, IQR = 12; range: 12–71). Likewise, CE comprehensiveness scores were significantly higher (W = 1764, *P* = 0·041) for ‘not-for-profit’ centres (median = 50/100, IQR = 28; range: 0–86) compared with ‘for-profit’ centres (median = 43/100, IQR = 11; range: 0–71). Results of the Kruskal–Wallis chi-squared test also indicated a significant effect of SEIFA index on CE strength scores (χ^2^(2) = 7·75, *P* = 0·021). Pairwise comparisons using Dunn’s test indicated that centres in the medium SEIFA index category had significantly lower CE strength scores (median = 14/100, IQR = 29; range: 0–43) than centres in both low (median = 29/100, IQR = 16; range: 0–57; Z = –2·13, *P* = 0·049) and high (median = 29/100, IQR = 24; range: 0–43; Z = –2·53, *P* = 0·034) SEIFA index categories. There was no difference observed in CE strength scores between centres in the high and low SEIFA index categories (Z = –0·53, *P* = 0·593). The zero-inflated negative binomial regression analysis for NS strength indicated a significant main effect of SEIFA index (χ^2^(2) = 6·01, *P* = 0·049). However, post hoc (Tukey) pairwise comparisons indicated no differences between any of the SEIFA categories (all *P*s > 0·05).

Table 3 shows WellCCAT item scores across the four domains. Within Domain 1 (NE), most policies did not address (scored 0) the allocation of funds for nutrition education (NE6, *n* 117). A majority of policies scored > 0 regarding provision of nutrition education for children (NE1, *n* 114), for educators (NE3, *n* 103) and for parents (NE4, *n* 110). Only one item was explicitly described by policies (score of 2) which referred to food-related activities being embedded in nutrition education (NE2, *n* 73).

**Table 3:** Childcare centre-based nutrition policy WellCCAT assessment scores

WellCCAT domains and constructs (*n* = centre-based nutrition policies)	Score of 0		Score of 1		Score of 2		Qualitative content analysis regarding application of WellCCAT to Australian centre-based nutrition policies
	*n*	%	*n*	%	*n*	%	
Domain 1 – Nutrition education							
NE1 – Provision of nutrition education for children	4	3 %	85	72 %	29	25 %	
NE2 – Food-related activities are consistent with nutrition education	14	12 %	31	26 %	73	62 %	
NE3 – Provision of nutrition education training for teachers	15	13 %	101	86 %	2	2 %	
NE4 – Provision of nutrition education for parents	8	7 %	103	87 %	7	6 %	
NE5 – Mealtime is used as an opportunity to teach nutrition concepts	23	20 %	89	76 %	5	4 %	
NE6 – Allocation of funds for nutrition education	117	99 %	1	1 %	0	0 %	Minor wording adjustment would increase score/additional policy document may address this item
Domain 2 – Nutrition standards							
NS7 – Nutrition standards go beyond Dietary Guidelines	1	1 %	116	98 %	1	1 %	Score of 2 appeared out of scope for childcare staff
NS8 – Nutrition standards for foods brought from home*	12	11 %	92	84 %	5	5 %	
NS9 – Replacing saturated fat with mono/polyunsaturated fats	114	97 %	4	3 %	0	0 %	Minor wording adjustment would increase score
NS10 – Providing whole-grain cereals	68	58 %	50	42 %	0	0 %	Score of 2 appeared out of scope for childcare staff
NS11 – Limiting sugar content of foods	21	18 %	95	81 %	2	2 %	
NS12 – Limiting Na content of foods	9	8 %	109	92 %	0	0 %	Score of 2 appeared out of scope for childcare staff
NS13 – Standards for portion sizes of foods	68	58 %	49	42 %	1	1 %	Question WellCCAT applicability/score of 2 appeared out of scope for childcare staff
NS14 – Providing both fruits and vegetables at lunch	22	19 %	95	81 %	1	1 %	Question WellCCAT applicability for strength score
NS15 – Providing fruits or vegetables for snack	20	17 %	98	83 %	0	0 %	
NS16 – Limiting fat content of milk for children aged 2 years and older	117	99 %	1	1 %	0	0 %	Minor wording adjustment would increase score
NS17 – Limiting the quantity of juice served	104	88 %	2	2 %	12	10 %	Minor wording adjustment would increase score
NS18 – Providing 100 % pure fruit or vegetable juice	106	90 %	0	0 %	12	10 %	Minor wording adjustment would increase score
NS19 – Serving food and beverages with no added sugar, artificial sweeteners, flavours, preservatives or enhancers	110	93 %	8	7 %	0	0 %	
NS20 – Limiting sugar-sweetened beverages	45	38 %	61	52 %	12	10 %	Minor wording adjustment would increase score
NS21 – Nutrition standards for flavoured milk	87	74 %	20	17 %	11	9 %	Minor wording adjustment would increase score
NS22 – Nutrition standards for celebrations	114	97 %	2	2 %	2	2 %	Additional policy document may address this item
NS23 – Nutrition standards for fundraising	87	74 %	31	26 %	0	0 %	Additional policy document may address this item
Domain 3 – Health promotion							
HP24 – Optimises scheduling of meals and snacks to improve child nutrition	13	11 %	105	89 %	0	0 %	Question WellCCAT applicability for a score of 2
HP25 – Ensures adequate time to eat	18	15 %	99	84 %	1	1 %	Question WellCCAT applicability for a score of 2
HP26 – Hand washing before meals	69	58 %	4	3 %	45	38 %	
HP27 – Providing a pleasant environment in which meals and snacks are eaten	1	1 %	23	20 %	89	79 %	
HP28 – Method for providing accessible drinking water throughout the day	1	1 %	74	63 %	43	36 %	
HP29 – Not pushing children to eat more than they want	9	8 %	75	64 %	34	29 %	
HP30 – Introducing children to a variety of foods	10	8 %	99	84 %	9	8 %	Minor wording adjustment would increase score
HP31 – Managing additional helpings of foods	95	83 %	14	12 %	6	5 %	Question WellCCAT applicability
HP32 – Teachers sit with children during meals	12	10 %	54	47 %	50	43 %	
HP33 – Teachers consume the same foods offered to children during meals	25	21 %	91	77 %	2	2 %	Question WellCCAT applicability for a score of 2
HP34 – Teachers assist children in gauging level of fullness	91	77 %	27	23 %	0	0 %	
HP35 – Specific course of action when food brought from home does not meet nutritional standards*	92	83 %	15	14 %	4	4 %	Additional policy document may address this item
HP36 – Food not being used as a reward and/or withheld as a punishment	18	15 %	16	14 %	84	71 %	
HP37 – Oversight of menu planning by a health professional	38	33 %	9	8 %	69	59 %	
HP38 – Provision of nutrition training for staff involved in cooking	35	30 %	67	57 %	16	14 %	
HP39 – Staff consumption of foods and beverages in front of children outside of mealtimes	71	60 %	47	40 %	0	0 %	Question WellCCAT applicability/appeared out of scope for ECE staff/additional policy document may address this item
Domain 4 – Communication and evaluation							
CE55 – Provides parents with referrals for health- and/or nutrition-related services	81	69 %	37	31 %	0	0 %	Question WellCCAT applicability
CE56 – Specifies marketing to promote healthy choices	113	96 %	4	3 %	1	1 %	Additional policy document may address this item
CE57 – Specifies restricting marketing of unhealthful choices	89	75 %	25	21 %	4	3 %	Additional policy document may address this item
CE59 – Provides written menus to parents	23	20 %	5	4 %	86	75 %	
CE60 – Addresses nutrition assessments of children	67	57 %	51	43 %	0	0 %	Question WellCCAT applicability for strength score
CE62 – Identifies a plan for evaluating or assessing health policies	71	61 %	10	9 %	36	31 %	Question WellCCAT applicability
CE63 – Identifies a plan for revising health policies	20	17 %	41	35 %	56	48 %	

CE58 and CE61 were removed as these items were considered out of scope being related to US-specific programs (e.g. Child and Adult Care Food Program).

* Indicates n/a responses possible (NS8 *n* 47; HP35 *n* 48).

A number of items within Domain 2 (NS) were infrequently addressed in policies (scored 0), namely, unsaturated fat provision (NS9, *n* 114), milk fat content (NS16, *n* 117), quantity of juice (NS17, *n* 104), quality of juice (NS18, *n* 106), added sugar and food additives (NS19, *n* 110), flavoured milk (NS21, *n* 87), celebrations (NS22, *n* 114) and fundraising (NS23, *n* 87). Almost all policies scored 1 when referring to the Australian Dietary Guidelines (NS7, *n* 117) and for limiting foods high in Na (NS12, *n* 109). The majority of policies scored 1 for the provision of both fruit and vegetables at lunch (NS14, *n* 95) and at snack-time (NS15, *n* 98). Few policies contained explicitly described content sufficient to score 2 for items within this domain.

Scores for items within Domain 3 (HP) were dispersed, with items most commonly not addressed (scored 0) being handwashing before meals (HP26, *n* 69), managing additional helpings (HP31, *n* 95), educators gauging a child’s level of fullness (HP34, *n* 91), managing food brought from home not meeting NS (HP35, *n* 92) and staff consuming foods/beverages in front of children outside of mealtimes (HP39, *n* 71). Almost all policies outlined a pleasant eating environment (HP27, *n* 117) and provided access to drinking water throughout the day (HP28, *n* 117). A majority of policies scored 1 regarding the scheduling of meals and snacks (HP24, *n* 105), ensuring adequate time to eat (HP25, *n* 99), introducing a variety of foods (HP30, *n* 99), educators consuming the same foods provided to children at meals (HP33, *n* 91) and provision of nutrition training for staff involved in cooking (HP38, *n* 67). Explicitly described content (scored 2) was frequently found in relation to providing a pleasant environment for eating (HP27, *n* 89), educators sitting with children during meals (HP32, *n* 50), food not being used as a reward or punishment (HP36, *n* 84) and oversight of menu planning by a health professional (HP37, *n* 69).

The majority of items within Domain 4 (CE) were not addressed (scored 0), namely, provision of health referrals for parents (CE55, *n* 81), marketing healthy choices (CE56, *n* 113), restricting marketing of unhealthy choices (CE57, *n* 89), nutrition assessment of children (CE60, *n* 67), and having an evaluation plan to assess ECE health policies (CE62, *n* 71). Two items were frequently comprehensively described (scored 1) – nutrition assessments of children (CE60, *n* 51) and having a plan to revise ECE health policies (CE63, *n* 41). One item was frequently explicitly described (scored 2) in relation to the provision of written menus to parents (CE59, *n* 86).

Qualitative content analysis resulted in four themes constructed from similar interpretations (by each researcher) of recurring reasons likely contributing to low WellCCAT scores. These themes were established around the applicability of WellCCAT for assessment of Australian CBNP, namely: (1) *question WellCCAT applicability* where a WellCCAT item may not be suitable for the Australian context (e.g. strict time schedules under the item ‘ensuring adequate time to eat’ HP25), (2) *out of childcare staff scope* where a WellCCAT item may be considered unrealistic and beyond the scope of educator knowledge (e.g. ‘limiting Na content’ NS12), (3) *minor wording adjustment* where small language adjustments within policies could alter a score from a 0 to a 2 (e.g. ‘flavoured milk standards’ NS21) and (4) *potential to be in an additional policy* where a WellCCAT item might be addressed in separate documents which may not be considered when assessing the nutrition policy *per se* (e.g. ‘celebration standards’ NS22) (see Table 3).

Additional data collected independent of the WellCCAT tool included nutrition policies referring to the existence of other policy documents (*n* 75) and the *Get Up and Grow* resources (*n* 68). Furthermore, some CBNP specifically mentioned offering a progressive meal service (*n* 52) or flexible mealtimes (*n* 37).

## Discussion

Our study describes the constructs of comprehensiveness and strength of written CBNP for a sample of Australian childcare centres. Findings indicate that policies generally had low scores, as defined by the WellCCAT tool, particularly low strength scores across all four WellCCAT domains. These findings provide important insights into areas where quality improvement of Australian CBNP could be made to support the implementation of best practice food provision and feeding guidance in ECE settings.

NE was the highest-performing domain in our study. This indicates that childcare centres are attempting to provide nutrition education through ‘hands-on’ activities such as cooking or gardening that are purposefully planned into the curriculum. These findings are consistent with NZ research that also found high NE scores when using WellCCAT to evaluate 114 policy documents from 31 childcare centres^(25)^. Health literacy is an important mechanism to support healthy dietary intake, and strengthening policy statements regarding nutrition education requirements in ECE settings could provide a conduit to enhancing health literacy for childcare staff and children.

Overall, centre policies in our study had low median comprehensiveness scores (60/100) and very low median strength scores (17/100). The comprehensiveness of a nutrition policy relates to the scope of the nutrition guidance (what is included in the content), and the strength relates to how well this content is described within the policies. These findings are similar to those reported by studies in the USA and NZ^(24,25)^ and indicate that not only is the scope of nutrition guidance subpar, but the language used to describe the policies may not be easily interpretable for childcare staff^(24,25)^. Our study therefore adds to the body of evidence regarding suboptimal written nutrition policies in childcare centres and extends this evidence to Australia^(31)^. This is of concern as CBNP may be the sole resource used by childcare staff to guide food provision and feeding practices.

Australian and international studies report that the existence of a CBNP does not necessarily lead to implementation and consequent practice change^(32,33)^ when they lack the detail needed to enhance implementation^(32–34)^. This is reflected in our study’s finding of low strength scores, indicating that CBNP lacked the language to effectively describe the guidelines. The vague or unclear language leaves nutrition guidance open to interpretation, and template policies sourced from nutrition professionals are one way to combat ineffective or unclear language^(35)^. However, centres that use policy templates provided by professionals may feel disconnected from the policy, and this may result in an inconsistency between policy and practice. Alternatively, it has been noted that centres that locally develop CBNP, tailored to individual centre needs, have increased understanding and in turn implementation of policy guidelines^(36)^. With research highlighting guideline interpretation in the childcare setting as a considerable barrier to best practice implementation, it is evident that clear, comprehensive and actionable CBNP guidelines are needed^(31,32,37)^.

The significantly lower scores in medium compared with low and high socio-economic areas for the CE domain were similarly observed in an NZ study where centres with a medium neighbourhood deprivation index had the lowest menu scores compared with centres with a low or high neighbourhood deprivation index^(38)^. Further investigation is warranted to understand this finding, for example, whether centres in lower socio-economic areas receive more government-level support and if centres in high socio-economic areas are already well-resourced, leaving centres in medium socio-economic areas centres with varying levels of support and accessibility. Alternatively, the difference in scores may also be attributable to the cross-sectional nature of the study. That is, although an association between medium socio-economic areas and low scores was identified in our study, it does not necessarily translate to causation.

Another significant finding was related to centre location, with rural centres scoring lower for the NE domain compared with metropolitan centres. Geographical location influences access to services, support and training, with greater access frequently available within metropolitan areas, which may explain this difference^(21)^. In relation to centre management type, not-for-profit centres had significantly higher comprehensiveness scores than for-profit centres for NS and CE domains. This may be attributable to the values and priorities of different types of centres, for example, not-for-profit centres are predominantly community-owned by community organisations, councils or parent committees with profits usually reinvested in the centre, whereas for-profit centres commonly share a percentage of profits with shareholders^(39,40)^. Future studies are needed to investigate location and socio-economic differences to identify how these determinants impact ECE nutrition environments.

Qualitative analysis identified limitations to the use of WellCCAT for the Australian context. A number of items appeared inconsistent with Australian childcare recommendations. For example, the criteria for item HP25 ‘Ensure adequate time to eat’ requires that for a score of 2, children should have at least 30 min for lunch and at least 20 min for breakfast. However, across all the Australian government resources including ACECQA^(19)^, *Get Up and Grow* ^(41)^ resources (which 68 out of the 118 policies referenced) and the Australian Dietary Guidelines^(42)^, a time requirement for meals is not provided^(41)^. Furthermore, the time stipulation in WellCCAT contradicts an emerging trend seen in childcare nutrition policies where food is offered using a progressive meal service which provides children with some autonomy over when they choose to eat and decide they’re hungry (within reason)^(43,44)^. This advice is also reflective of the shifting evidence since WellCCAT was developed where child-feeding practices have moved towards responsive feeding principles^(45,46)^. In our study, 52 of 118 CBNP referred specifically to progressive meal service, and a further 37 stated they would be ‘flexible’ with meal duration. In future applications, policy analysis should consider scoring positively for flexible food provision rather than for prescriptive meal lengths. An additional example relates to items NS14 and NS15, regarding the combined provision of fruits and vegetables, and scoring implies that either may be served for lunch or snacks. Australia has separate recommendations for these food groups, and data show that substantially fewer children consume recommended vegetable intakes (6 %)^(47)^ compared with fruit intakes (73 %); therefore, vegetables need more emphasis in health promotion activities^(47)^.

Another limitation of WellCCAT is related to instances of unreasonable expectations of childcare staff. For example, item NS12 regarding the Na content of food, required ‘a quantified limit of less than 600 mg of Na per 100 g’ to be written into the CBNP to achieve a score of 2. This seems out of scope of the role of childcare staff to be able to quantify and calculate the Na content of foods, extrapolating ingredients to per 100 g prepared food, without nutrition training. Existing literature highlights that childcare staff face many challenges in the workplace with high levels of stress or long work hours; thus it is critical to ensure that policy actions do not add an unreasonable burden^(48,49)^. A 2020 systematic review (*n* 21 studies) adds that whilst policies may be in place, one reason they are not implemented either correctly or at all is due to written language not being clear enough to assist staff on how to apply these NS^(32)^. Therefore, if centres are seemingly already struggling to implement basic standards outlined in policies, including complex NS may exacerbate the issue. Information within a childcare nutrition policy therefore needs to be feasible.

In a number of instances, it was apparent that policies needed to be more explicit in the language used, most often in relation to the NS domain. For example, two policies which scored highest simply stated that milk and water were the only beverages served in the centre. These policies automatically scored 2 for five items within the NS domain relating to other beverages (fruit/vegetable juices, flavoured milk, sugar-sweetened) as the policies explicitly prohibited beverages other than unflavoured milk and water. Nearly all policies (*n* 117) referred to the Australian Dietary Guidelines (score of 1); however, language rarely went beyond this.

An interesting finding in our study was the degree of cross-referencing in CBNP (*n* 75) to additional policies and resources, such as parent and staff handbooks, infant feeding guidelines, food allergy policy and celebration policy. WellCCAT items addressing fund allocation (NE6), celebrations (NS22), fundraising (NS23), staff eating habits (HP39) and food marketing (CE57), which scored minimally in this analysis, may have been present in these separate documents which were not accessed for our study. A 2015 NZ study reported identifying additional policy documents (*n* 11) when assessing written nutrition policies from 112 childcare services^(25)^. Centres may therefore have further information that could potentially lead to higher WellCCAT scores but are not mentioned in their CBNP^(25)^. This is especially the case for item N22 that addresses NS for celebrations. The majority of CBNP in our study (*n* 114) scored 0 for this item though stronger guidelines may have been included in a separate celebrations policy. A concern here is the provision of discretionary foods as part of celebrations, as reported by Gerritsen *et al*., (2015) where one in four services provided three or more discretionary choices (high sugar, saturated fat or salt) on special occasions^(25)^. It may be argued that all relevant information should be contained in a single policy so that childcare staff can access required nutritional information and guidance in a centralised manner. This was the expectation of our study, requesting the upload of a single nutrition policy document. However, if centres prefer to distribute nutrition information across a selection of documents, then future research studies need to be designed to enable the collection of this additional information.

Due to the initial low participation, only nineteen policy documents were provided by centres, and the remaining ninety-nine were sourced from publicly available centre websites. Analysis of policies collected using two methods may present a bias in that online policies may not be the most recent policy adopted by the centre. However, given online policies were publicly accessible, these documents were considered appropriate to include. Further investigation into CBNP should aim to include a larger sample and a consistent data collection method to ensure a greater representation of centres.

The high representation of rural (45 %) and low SEIFA (35 %) centres was a strength of our study as it enabled the exploration of centre characteristics that have been attributed to health disparities among population groups. Moreover, the overlay of qualitative content analysis allowed for a more in-depth exploration of the nutrition policy assessments which had not been previously considered and resulted in a greater understanding and interpretation of study findings.

Whilst nutrition policy represents only one aspect of the overall childcare environment, it has the capacity to impart long-lasting health benefits to young children. Addressing the inadequacy of CBNP is likely to be one crucial step towards aligning childcare food provision and feeding behaviours with best practice guidelines^(31)^. However, opportunities for improvement should not be solely limited to policy. A multi-level approach would be beneficial, including appropriate nutrition training of childcare staff, assistance to centres in developing high-quality, actionable nutrition policies, current and contemporary national guidelines, national menu planning guidelines for consistent implementation across jurisdictions and a country-specific policy assessment tool.

### Conclusion

This study is the first in Australia to explore the comprehensiveness and strength of CBNP in Victorian childcare, making a unique contribution to the literature. It highlights the relatively low performance of such policies, regardless of centre location, management type or socio-economic area. Study findings highlight the necessity to consider the development of a country-specific tool to evaluate CBNP, thereby providing meaningful contextual data to enhance implementation of best practice food provision or feeding behaviours within ECE settings.
