# Nutrition environments in early childhood education: do they align with best practice? – ERRATUM

**DOI:** 10.1017/S1368980024001277

**Published:** 2024-08-13

**Authors:** Anna Aristova, Alison C Spence, Christopher Irwin, Audrey Elford, Laura Graham, Penelope Love

During copyediting of this article the abbreviation ‘CNBP’ was inadvertently changed to ‘CBNP’ in multiple locations. This has since been corrected.

Cambridge University Press apologise for this error.
